# Supplementary material for: Asymmetric Synthesis of Quaternary Hydantoins via a Palladium-Catalyzed Aza-Heck Cyclization
Source: J Am Chem Soc. 2025 Nov 14;147(49):44692–8. doi: 10.1021/jacs.5c16022 (PMC12703750; doi:10.1021/jacs.5c16022)
Supplement: Supplementary file 2 [file ja5c16022_si_002.zip › All NMR FID Files/17/17_ProtonNMR/1/pdata/1/email_EM07-005-2-dry-1H_1_1.pdf]

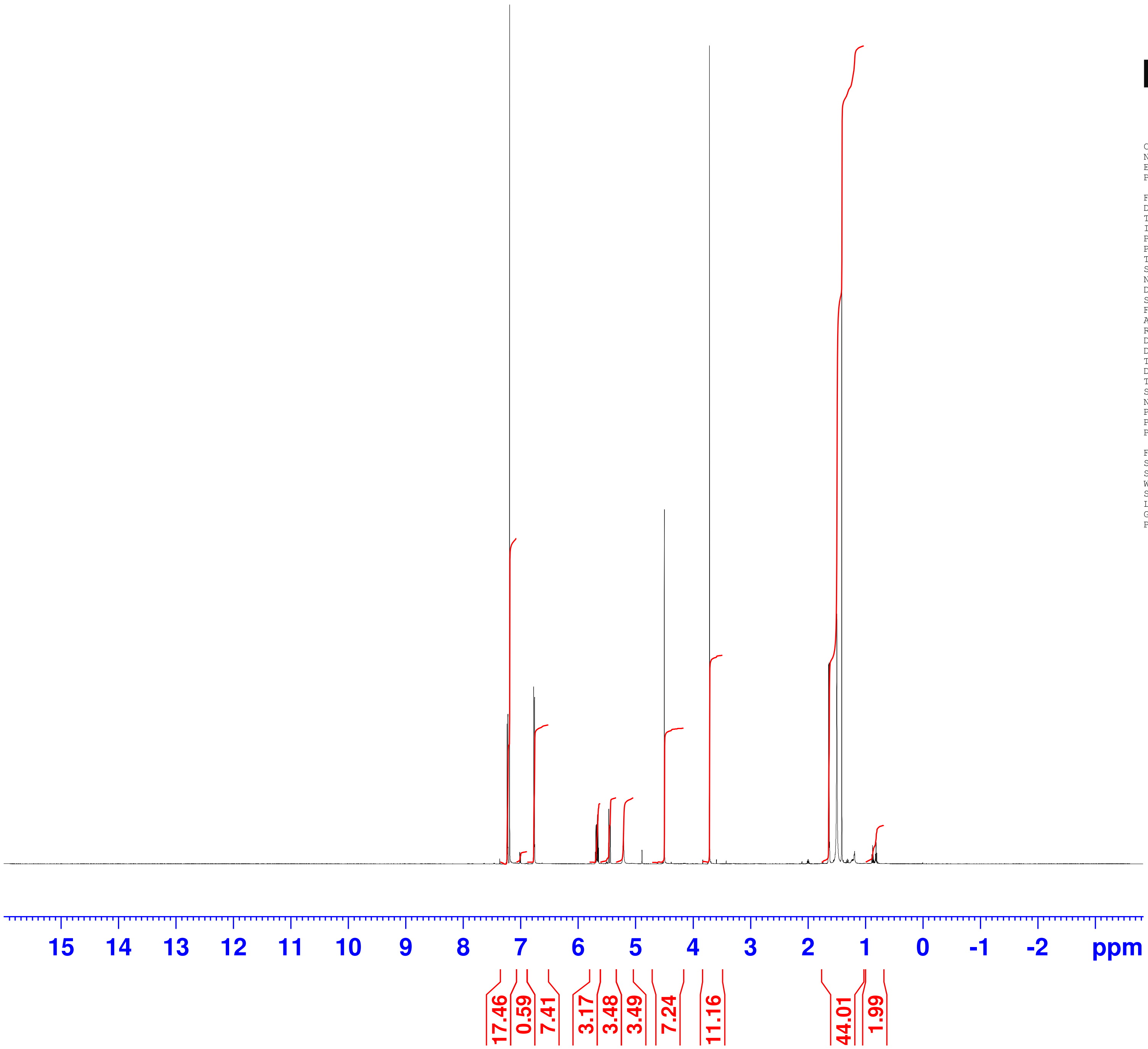


```
Current Data Parameters
NAME      EM07-005-2-dry-1H
EXPNO                     1
PROCNO                    1

F2 - Acquisition Parameters
Date_              20250911
Time                  13.28 h
INSTRUM  Avance Neo 600 at University of Delaware
PROBHD   Z185170_0001 (
PULPROG                zg30
TD                    65536
SOLVENT               CDCl3
NS                       16
DS                        2
SWH              11904.762 Hz
FIDRES            0.363304 Hz
AQ               2.7525120 sec
RG                     20.2
DW                   42.000 usec
DE                    14.30 usec
TE                    299.1 K
D1             1.00000000 sec
TD0                       1
SFO1         600.1337058 MHz
NUC1                     1H
P0                     2.83 usec
P1                     8.50 usec
PLW1           6.90000010 W

F2 - Processing parameters
SI                    65536
SF           600.1300566 MHz
WDW                      EM
SSB                       0
LB                     0.30 Hz
GB                        0
PC                     1.00
```
